# Relationship between Standing Trunk Extension Angle and Medial Elbow Injuries in Young Baseball Pitchers

**DOI:** 10.3390/ijerph19073895

**Published:** 2022-03-24

**Authors:** Megumi Gonno, Noriyuki Kida, Teruo Nomura, Tomoyuki Matsui, Yoshikazu Azuma, Machiko Hiramoto, Ruo Hashimoto, Tetsuya Miyazaki, Maki Tanaka, Yuya Watanabe, Yoshihiro Kai, Toru Morihara

**Affiliations:** 1 Graduate School of Science and Technology, Kyoto Institute of Technology, 1 Hashikami-cho, Matsugasaki, Sakyo-ku, Kyoto 606-8585, Japan; m-tanaka@po.kbu.ac.jp; 2 Faculty of Arts and Sciences, Kyoto Institute of Technology, Kyoto 606-8585, Japan; kida@kit.ac.jp (N.K.); nomura@kit.ac.jp (T.N.); 3 Marutamachi Rehabilitation Clinic, Kyoto 604-8405, Japan; matsui.tomoyuki.sports.reha@gmail.com (T.M.); azuma.yoshikazu.reha@gmail.com (Y.A.); true.to.your.heart810@gmail.com (M.H.); ruo.hashimoto@gmail.com (R.H.); mtsports0512@gmail.com (T.M.); toru4271@koto.kpu-m.ac.jp (T.M.); 4 Physical Fitness Research Institute, Meiji Yasuda Life Foundation of Health and Welfare, Tokyo 100-0005, Japan; yu15-watanabe@my-zaidan.or.jp; 5 Department of Physical Therapy, Faculty of Health Sciences, Kyoto Tachibana University, Kyoto 607-8175, Japan; kai-y@tachibana-u.ac.jp

**Keywords:** junior baseball player, elbow injury, trunk extension, thoracic extension

## Abstract

Purpose: The purpose of this study was to investigate the relationship between the standing trunk extension angle and medial elbow injuries. Subjects and methods: The study participants were 90 male baseball pitchers (10–12 years) belonging to youth baseball teams. Pitching elbow injuries were evaluated by an orthopedic surgeon using ultrasound scans and physical examination findings. A single optical three-dimensional motion analysis system was used for the trunk extension measurements, with three-dimensional coordinates captured. The overall, upper, and lower trunk angles were then analyzed. Results: Trunk extension angle during standing trunk extension was significantly smaller among participants who were positive for medial elbow injuries on ultrasound scans (positive: 71.4° ± 10.3°; negative: 75.7° ± 9.2°; *t* = 2.05, *p* < 0.05). The upper trunk extension angle was significantly smaller than the lower trunk extension angle among participants who were positive for medial elbow injuries on physical examination (upper: 33.0° ± 6.9°; lower: 41.2° ± 8.2°; *t* = −2.42, *p* < 0.05). Conclusions: Trunk extension angle during standing trunk extension is associated with medial elbow injuries. Evaluating the trunk extension angle as multiple segments rather than a single rigid body is valuable.

## 1. Introduction

Baseball players often suffer pitching-related shoulder or elbow injuries, which may halt a competitive career if a serious injury occurs during the athlete’s growth phase. Medial elbow injuries are common in youth baseball players. Risk factors for pitching-related elbow injuries include age, number of years as a pitcher, defensive position, total number of yearly appearances, frequency of weekly training, and daily pitch count [1,2,3,4,5]. Parameters related to the frequency of pitching are thus the most important factors of pitching-related elbow injuries.

In addition to the amount of pitching, other factors shown to be associated with pitching-related elbow injuries include shoulder external rotator and internal rotator muscle strength, shoulder internal rotator muscle tightness, shoulder overall rotation angle (the sum of the internal and external rotation angles), shoulder flexion angle, shoulder internal rotation angle, shoulder external rotation angle, thoracic kyphosis angle, and elbow extension deficit [3,4,6,7,8]. 

The valgus stress experienced in the elbow joint during pitching can be affected by elbow joint range of motion (ROM) and surrounding muscle strength that may increase or decrease the risk of elbow injuries. Valgus stress on the elbow is at its greatest at shoulder maximum external rotation (MER) in the acceleration phase of the pitching action, and the ratio of the MER to the passive ROM of external rotation of the throwing shoulder has been found to be significantly greater in baseball players with a history of elbow injury [9]. This finding reflects the role that external rotation ROM of the shoulder has in contributing to valgus elbow stress.

The MER of the shoulder during the acceleration phase of the pitching action is affected by the external rotation of the glenohumeral joint, scapulothoracic joint movement, and thoracic spine extension [10]. Finley and Lee [11] showed that when thoracic spine kyphosis increases, there are significant decreases in the posterior tip and lateral rotation of the scapula. Fleisig et al. [12] pointed out that the kinetic chains of the glenohumeral joint, scapulothoracic joint, and trunk play an important role in pitching performance and injury prevention. That is, the external rotation of the shoulder during the pitching action is achieved not solely as a result of the external rotation of the scapulohumeral joint, but also by the trunk and upper arm as a whole, including the movements of the scapulothoracic joint and thoracic spine. Therefore, the extension ROM of the trunk is believed to affect the external rotation of the shoulder and contributes to valgus stress on the elbow during pitching.

Sakata et al. [2] showed that static standing thoracic kyphosis is associated with medial elbow injuries. In that study, measurements were taken in the stationary standing position, but alignment in the stationary standing position may differ from ROM while pitching. A study of junior high and high school students [13] found that the static standing thoracic kyphosis angle was unrelated to the incidence of medial elbow injuries. However, the magnitude of the change in the angle of the thoracic spine when the arms were raised after standing still was smaller in those subjects with elbow injuries. This suggested that it may be more appropriate to measure ROM associated with trunk movements. Therefore, in this study, we focused on measuring trunk extension ROM as a method of evaluating thoracic extension.

Trunk extension ROM, which measured the angle between the basic axis as the posterior surface of the sacrum and the movement axis as a line joining Th1 and L5, with the trunk regarded as a single rigid body, is generally considered to be 30° [14]. However, to study the trunk ROM of baseball players, it is important to assess thoracic extension by regarding the trunk as three rigid segments comprising the thorax, lumbar, and pelvis, rather than as a single rigid body, and measuring the angle between the thorax and the lumbar. In studies that have evaluated the extension movement of the thorax with the trunk treated as two rigid segments, reflective markers were attached to C7, Th8, and L1 and the angle of thoracic extension during pitching action was measured; the angle of thoracic extension at MER was shown to be about 10–20° [10,15]. This angle does not include the ROM of the lumbar. The curvature of the spine and spinal movement are most accurately evaluated by X-rays. However, due to radiation exposure during X-rays, measurement methods that can be used on the surface of the body have been developed. Kyphometers and goniometers can only measure one angle at a time [16], and although the spinal mouse is highly accurate when used in stationary postures, its accuracy diminishes during trunk flexion or extension [17]. If a three-dimensional (3D) motion analysis system is used, the posterior surface of the body is likely to be in the camera’s blind spot when the trunk is in an extended posture.

Elbow pain and discomfort have been studied using a variety of methodologies, including interviews and questionnaires, but in this study ultrasound scans and physical examination will be used. Ultrasound scanning is widely used for the evaluation of elbow injuries and to assess morphological abnormalities of the bones and soft tissues in the elbow. Harada et al. [18] investigated the reliability of ultrasound scans in detecting abnormalities by comparing scans of 25 young baseball players with X-rays and showed that ultrasonography is useful for the detection of elbow injuries. Among pitching-related elbow injuries, ultrasound scanning is capable of identifying a previous elbow injuries caused by pitching irrespective of whether the elbow is painful when the scan is performed. Physical examination, on the other hand, evaluates pain at the time of examination, and positive detection of elbow injuries increases when it is conducted on the field immediately after a match or practice session. The timing of the investigation also affects the injury rate, which increases during the baseball season and decreases in the off-season. We used both ultrasound scans and physical examinations to evaluate medial injuries to the elbow and investigated previous stress and acute pain on the elbow separately. 

The purpose of this study was to investigate the relationship between the standing trunk extension angle and medial elbow injuries. The novel aspects of the study are that (1) dynamic trunk extension angle is measured, (2) the trunk angle is measured separately for the upper and lower trunk, and (3) both ultrasound scans and physical examinations are used. The study was designed as a cross-sectional case-control study.

## 2. Material and Methods

### 2.1. Study Participants

The study participants were 90 male baseball pitchers of elementary-school age (10–12 years, comprising 82 fifth-grade and 8 sixth-grade elementary school students) belonging to youth baseball teams. Written informed consent was obtained in advance from the study participants and their parents or guardians. This study was also conducted with the approval of the Human Research Ethics Committee of Kyoto Institute of Technology.

The mean height of the study participants was 144.6 ± 7.2 cm and their mean weight was 36.9 ± 6.7 kg (Table 1). The differences between the mean heights and weights of the fifth-graders and sixth-graders were tested but were not found to be statistically significant.

A questionnaire survey was used to investigate the amount of practice, the frequency of training sessions per week, time spent training on weekends and national holidays, and the maximum full-strength pitch count on a single day during the previous week. The frequency of training sessions per week was scored on a five-point scale from one to seven times, the time spent training on weekends and national holidays was scored on a five-point scale from less than 4 h to 7 h or more, and the maximum full-strength pitch count on a single day during the previous week was scored on an eight-point scale from less than 40 pitches to 100 pitches or more (Table 2). These categories of survey questions and response choices were designed specifically for this study and were based on those used in previous studies [2,3,4,19].

### 2.2. Measurements

(a) Evaluation of pitching elbow injuries

Pitching elbow injuries was evaluated by an orthopedic surgeon using ultrasound scans and physical examination findings. Any study participant with an irregularity, separation, or protrusion visible on the ultrasound scan was classed as positive for pitching elbow injuries [18]. Any study participant who exhibited tenderness of the medial elbow or valgus stress in the physical examination was also classed as positive for pitching elbow injuries.

(b) Thoracic spine angle measurements

The thoracic kyphosis angle was evaluated by measuring the angles of inclination formed between the spinous processes of Th1–2 and the spinous processes of Th12–L1 with an inclinometer to an accuracy of 1°. These two angles were then added to obtain the thoracic kyphosis angle, and flexed position was defined as positive [2,20].

(c) Trunk extension angle measurement

**Figure 1:**
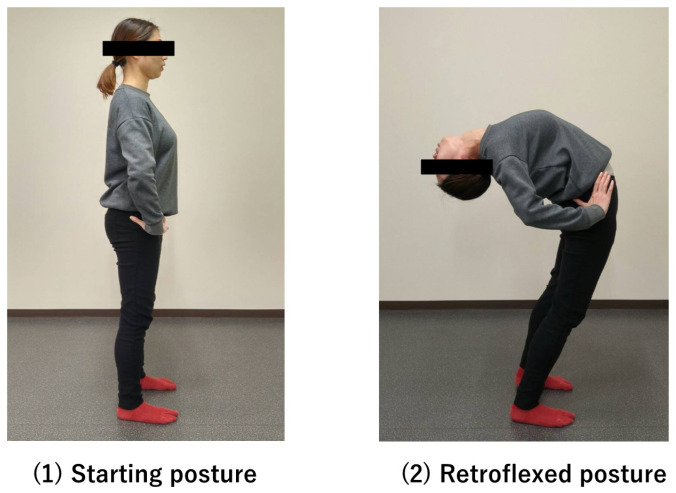
Instructions for trunk extension angle measurement: From the starting position, bend over backwards and hold a position of maximum extension for 3 s.

In trunk extension measurements, the participants first stood with their legs shoulder-width apart and their hands placed on their lower back. From this posture, they were instructed to bend their upper body backward while keeping the knees extended until they achieved maximum extension and to maintain this position for three seconds (Figure 1). Before starting, the participants were given a demonstration and an oral explanation of the procedure and they practiced it two to three times before measurements were taken.

**Figure 2:**
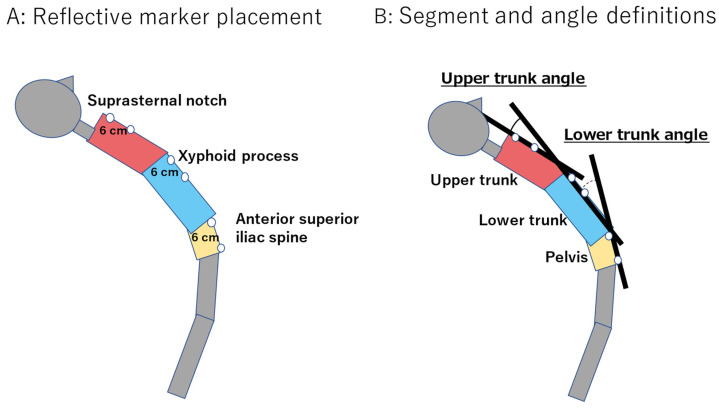
(**A**) Marker positioning for trunk extension angle measurements; (**B**) Definitions of angles and segments.

A single optical 3D motion analysis system (OptiTrack V120 DUO, NaturalPoint Inc., Corvallis, OR, USA) was used for all the measurements, with 3D coordinates captured by using reflective markers placed on the front of the body [21]. Each of the three sets of two reflective markers was fixed by tape at a distance of 6 cm from each other. These marker sets were affixed to the suprasternal notch, the xyphoid process, and the right anterior superior iliac spine. The straight line formed by the two markers from the suprasternal notch was defined as the upper trunk, the straight line formed by the two markers from the xyphoid process was defined as the lower trunk, and the straight line formed by the two markers from the right anterior superior iliac spine was defined as the pelvis. The angle formed by the intersection of the upper trunk and lower trunk was defined as the upper trunk angle, and the angle formed by the intersection of the lower trunk and the pelvis as the lower trunk angle (Figure 2). The overall trunk angle was defined as the sum of the upper and lower trunk angles. We analyzed upper trunk angle, lower trunk angle, and overall trunk angle defined as the sum of the upper and lower angles.

### 2.3. Statistical Analysis

We first used a χ^2^ test to investigate the difference in the incidence of pitching elbow injuries between the two school grades. A Mann-Whitney U test was used to compare the incidence of pitching elbow injuries according to the amount of training. Pearson’s correlation coefficient was calculated to investigate the association between trunk angle and age, number of years of competitive baseball, physique, and thoracic spine angle. A *t*-test was used to investigate differences in the incidence of pitching elbow injuries according to age, number of years of competitive baseball, physique, thoracic spine angle, and trunk angle. A *t*-test was also used to investigate differences in upper and lower trunk angles depending on the presence or absence of ultrasound findings and the results of physical examination. Statistical analysis was conducted by using statistical software (IBM SPSS 27). We used an alpha level of 0.05 for all statistical tests.

## 3. Results

### 3.1. Pitching Elbow Injuries and Amount of Training

Physical examination identified 13 participants who were positive for elbow injuries (14.4%) and 77 who were negative (85.6%). By school grade, the positive participants comprised 12 fifth-graders (14.6%) and 1 sixth-grader (12.5%), with no significant difference in incidence between the two grades (χ^2^ = 0.027, n.s.). Ultrasound scans identified 51 positive participants (56.7%) and 39 who were negative (43.3%). By school grade, the positive participants comprised 46 fifth-graders (56.1%) and 5 sixth-graders (62.5%), with no significant difference in incidence between the two grades (χ^2^ = 0.122, n.s.). The participants who were identified as positive by ultrasound comprised 11 (84.6%) of those who were positive on physical examination and 40 (51.9%) of those who were negative on physical examination. The participants who were identified as positive by physical examination comprised 11 (21.6%) of those who were positive on ultrasound and 2 (5.1%) of those who were negative on ultrasound. There was a significant correlation between the results of physical examination and ultrasound findings (χ^2^ = 4.834, *p* < 0.05).

A Mann-Whitney U test was used to compare the incidence of pitching elbow injuries according to the amount of training (Table 2). There was a significant difference in the results of physical examination depending on the maximum full-strength pitch count on a single day during the previous week (*U* = 689.0, *p* < 0.05), with a higher count associated with a significantly higher rate of pitching elbow injuries. However, there was no significant difference in terms of ultrasound findings.

**Table 2:** Numbers and percentages of participants with signs of injury (frequency of training, duration of training, pitch count).

	Physical Signs						Ultrasound Signs					
	Positive		Negative				Positive		Negative			
	*n*	%	*n*	%	*U*	*p*	*n*	%	*n*	%	*U*	*p*
Weekly frequency of training					407.5	0.226					948.5	0.671
1 day	0	0.0%	0	0.0%			0	0.0%	0	0.0%		
2 days	9	69.2%	37	48.1%			27	52.9%	19	48.7%		
3–4 days	3	23.1%	37	48.1%			22	43.1%	18	46.2%		
5–6 days	1	7.7%	1	1.3%			1	2.0%	1	2.6%		
7 days	0	0.0%	2	2.6%			1	2.0%	1	2.6%		
Time spent training on weekends and national holidays					491	0.910					1063	0.566
<4 h	1	7.7%	1	1.3%			1	2.0%	1	2.6%		
<5 h	0	0.0%	13	16.9%			7	13.7%	6	15.4%		
<6 h	5	38.5%	15	19.5%			13	25.5%	7	17.9%		
<7 h	3	23.1%	24	31.2%			11	21.6%	16	41.0%		
≥7 h	4	30.8%	24	31.2%			19	37.3%	9	23.1%		
Full-strength pitch count in one day					689	0.027 *					1218	0.063
<40	2	15.4%	28	36.4%			14	27.5%	16	41.0%		
<50	2	15.4%	13	16.9%			8	15.7%	7	17.9%		
<60	0	0.0%	9	11.7%			3	5.9%	6	15.4%		
<70	2	15.4%	8	10.4%			9	17.6%	1	2.6%		
<80	2	15.4%	7	9.1%			5	9.8%	4	10.3%		
<90	2	15.4%	6	7.8%			5	9.8%	3	7.7%		
<100	0	0.0%	2	2.6%			1	2.0%	1	2.6%		
≥100	3	23.1%	4	5.2%			6	11.8%	1	2.6%		

* *p* < 0.05.

### 3.2. Trunk Extension Angle

Table 3 shows the mean age in months, years of competitive baseball, physique, thoracic spine angles, and trunk angle and their correlation coefficients of each variable with the trunk angle. Overall trunk angle was significantly correlated with both the upper and lower trunk angles (upper trunk angle: *r* = 0.446, *p* < 0.01; lower trunk angle: *r* = 0.752, *p* < 0.01), and there was also a significant negative correlation between the upper and lower trunk angles (*r* = −0.233, *p* < 0.05). Height was also significantly negatively correlated with both the overall trunk angle and upper trunk angle (overall trunk angle: *r* = −0.228, *p* < 0.05; upper trunk angle: *r* = −0.272, *p* < 0.05).

### 3.3. Relationship with Pitching Elbow Injury

We investigated the differences in the incidence of positive findings according to the mean values of the between-subject factors of age in months, years of competitive baseball, physique, thoracic spine angles, and trunk angle (Table 4). The results showed no significant difference for any factor in terms of the results of physical examination, but the participants found to be positive by ultrasound were significantly older (positive: 139.0 ± 5.0 months; negative: 136.6 ± 4.9 months; *t* = 2.27, *p* < 0.05). The overall trunk angle was also significantly smaller for positive participants (positive: 71.4° ± 10.3°; negative: 75.7° ± 9.2°; *t* = 2.05, *p* < 0.05). We then investigated the upper and lower trunk angles separately and found that in participants who were positive on physical examination, the lower trunk angle was significantly greater than the upper trunk angle (*t* = −2.42, *p* < 0.05), whereas for those who were negative no such significant difference was evident *(t* = −1.35, n.s.). There was no significant difference between the upper and lower trunk angles among participants who were either positive or negative on ultrasound scans (positive: *t* = −1.69, n.s.; negative: *t* = −1.30, n.s).

## 4. Discussion

### 4.1. Trunk Extension Angle

In this study, 14.4% of the participants were found to be positive on physical examination and 56.7% by ultrasound scans. Of those who were found to be positive by ultrasound, 78.4% were negative on physical examination. Sakata et al. [2] reported that around 20% of participants were positive by ultrasound. The figures in this study were influenced by the fact that our study participants were all pitchers, who therefore threw the ball more often compared with players in other positions. That previous study also excluded participants who had previously suffered elbow pain, which was not the case in this current study, and this may have contributed to the lower positive rate for ultrasound findings. With respect to the amount of training in the previous week, there was no significant difference in the frequency of training sessions per week or time spent training on weekends and national holidays by participants who were positive on physical examination compared with those who were negative, but their maximum full-strength pitch count on a single day was higher. This confirmed that participants who exhibited physical signs had placed the elbow under valgus stress by excessive pitching, resulting in pain. However, there was no difference between the positive participants and the negative participants in terms of ultrasound findings. Participants who were positive by ultrasound scans exhibited morphological abnormalities of the bone on the medial side of the elbow despite not having placed the elbow under excessive stress immediately prior to measurements. This result confirmed that the elbow had not been placed under excessive valgus stress due to overpitching immediately prior to measurements, but at a time before this. Thus, ultrasound scans are able to identify previous injuries to the elbow.

In this study, participants found to have medial elbow injuries by ultrasound had a smaller trunk extension angle compared with those found to be negative. This result suggests that the magnitude of the trunk and thoracic extension ROM may alleviate the valgus stress placed on the elbow by the pitching action, a result consistent with the findings of previous studies [2,10,13]. However, Saito et al. [22] reported that players with elbow injuries had smaller hip joint ROM. Shitara et al. [23,24] also showed that players with smaller pre-season passive shoulder external rotation ROM in 90° abduction and ankle ROM tended to be more susceptible to medial elbow injuries. Diminished ROM of the arms and legs as well as of the trunk are thus associated with elbow injuries. Accordingly, although in this study we found an association between trunk extension angle and ultrasound findings, the possibility that what was observed was a reduced trunk extension angle due to poorer overall flexibility cannot be excluded.

In this study, we did not observe an association between the static thoracic kyphosis angle and the trunk extension angle. There was also no significant difference between the static thoracic kyphosis angle of participants who were identified as positive on physical examination or ultrasound scans. In this study, we measured the kyphosis angle of the thoracic spine following the method of Sakata et al. [2] which reported that a static thoracic kyphosis angle exceeding 30, that is, a forward-bending posture when standing, was associated with medial elbow injury, but the results in our study were inconsistent with that finding. The detection rate may have been higher because dynamic measurements are affected by muscle strength as well as by flexibility and impose a physical burden closer to that of actual pitching movements.

### 4.2. Physical Signs and Trunk Extension Angle

There was no difference in the overall trunk extension angle between participants who were positive or negative on physical examination. However, in the positive participants, the upper trunk angle was significantly smaller than the lower trunk angle. This characteristic was only observed in participants who were positive on physical examination. Decreased thoracic extension ROM can thus be described as an important characteristic of pitchers with elbow injuries evident on physical examination, and study participants with elbow pain also had reduced thoracic extension ROM in the trunk. This result suggests that thoracic extension ROM contributes to elbow stress. Those study participants who were positive on physical examination also had a higher maximum full-strength pitch count on a single day during the previous week. Sakata et al. [25] compared youth baseball players who underwent a pitching injuries prevention program with a control group and found that the thoracic kyphosis angle improved and that shoulder and elbow injury rates were significantly lower in the intervention group compared with the control group. This means that engaging in conditioning and improving thoracic extension ROM might be possible to help prevent the occurrence of medial elbow injuries. The participants in the present study may have engaged in excessive full-strength pitching while in a state of diminished extension ROM of the thorax, placing the elbow under excessive stress and causing pain. This result could not have been obtained if the trunk had been regarded as a single rigid body and the angle formed between the legs and the trunk had been taken as the angle of extension, as the difference between the upper and lower trunk angle could not have been evaluated. Our study thus showed the possibility of evaluating the trunk as multiple rigid segments rather than as a single rigid body. 

### 4.3. Limitations and Future Research

This study had the following four limitations. First, we observed a negative correlation between trunk extension angle and height. Standing trunk extension is known to impose a severe load on the lumbar. Accordingly, investigations using the method of measuring the standing trunk extension angle adopted in this study are limited in terms of factors such as the age, sporting experience, and type of sport that can be addressed. Second, the thoracic kyphosis angle in stationary standing was measured as the angle of the spinal column from the back of the body. The standing trunk extension angle, however, was measured from the front of the body, thus comparisons of the two may be problematic. Third, an association was observed between trunk extension angle and medial elbow injuries. However, this study may have had a Type I error due to repeated testing. Therefore, caution should be exercised in interpreting the results of the present study. In addition, as this was a cross-sectional study, the cause of the injuries or pain was not identified. It is impossible to distinguish precisely whether restricted ROM of the thorax caused excessive stress on the elbow, or whether a situation that resulted in excessive stress on the elbow also reduced the ROM of the thorax. Further studies including longitudinal surveys and interventional studies are required to ascertain the cause. Fourth, we analyzed the angle of extension during maximum trunk extension in standing, which meant that we analyzed still images of dynamic ROM. When investigating connections with injuries or pain, rather than looking solely at the magnitude of ROM, it is important to bear in mind the fact that “whiplash” movements may occur depending on the starting position for the movement and kinetic chains [10]. Undertaking further studies using video for motion analysis may elucidate the characteristics in greater detail.

Despite the above issues and limitations, this is a significant study that demonstrates novelty and value in respect of the following points. Flexion-extension movements of the trunk also involve the hips and legs. Standing trunk extension, the movement addressed in this study, may also be associated with hip or thigh injuries. Dividing the trunk into several segments and categorizing them by their individual characteristics during standing trunk extension may also be useful for the early discovery of elbow and lumbar injuries.

## 5. Conclusions

Trunk extension angle during standing trunk extension was significantly smaller among participants who were positive for medial elbow injuries on ultrasound scans (positive: 71.4° ± 10.3°; negative: 75.7° ± 9.2°; *t* = 2.05, *p* < 0.05). The upper trunk extension angle was significantly smaller than the lower trunk extension angle among participants who were positive for medial elbow injuries on physical examination (upper: 33.0° ± 6.9°; lower: 41.2° ± 8.2°; *t* = −2.42, *p* < 0.05). Trunk extension angle during standing trunk extension was associated with medial elbow injuries. Evaluating the trunk extension angle as multiple segments rather than a single rigid body was valuable.

## Figures and Tables

**Table 1:** Participants’ height and weight.

	Fifth Grade (*n*= 82)	Sixth Grade (*n* = 8)	*t*	*p*
Height (cm)	144.6 ± 7.1	145.0 ± 8.8	−0.15	0.88
Weight (Kg)	36.9 ± 6.8	37.6 ± 5.8	−0.30	0.77

**Table 3:** Associations between basic attributes, thoracic spine angles, and trunk angles.

		Association with Trunk Angles					
		Overall Angle		Upper Angle		Lower Angle	
	*M* ± *SD*	*r*	*p*	*r*	*p*	*r*	*p*
Basic attributes							
Age (months)	138.0 ± 5.1	−0.051	0.635	−0.116	0.277	0.031	0.775
Number of years of competitive baseball	3.8 ± 1.1	0.101	0.344	0.177	0.096	−0.021	0.847
Number of years pitching	2.0 ± 1.1	−0.054	0.610	0.073	0.494	−0.114	0.283
Height (cm)	144.6 ± 7.2	−0.228	0.031 *	−0.272	0.009 **	−0.048	0.654
Weight (kg)	36.9 ± 6.7	−0.036	0.737	−0.192	0.070	0.104	0.331
Static standing thoracic angles							
Kyphosis angle (°)	29.6 ± 7.9	−0.004	0.971	0.071	0.507	−0.057	0.593
Thoracic spine angle Th1 (°)	18.0 ± 5.9	0.024	0.820	−0.078	0.470	0.085	0.430
Thoracic spine angle Th12 (°)	11.6 ± 5.2	−0.022	0.838	−0.021	0.847	−0.009	0.936
Standing trunk retroflexion angles							
Overall angle (°)	73.2 ± 10.0	-		0.446	0.000 **	0.752	0.000 **
Upper angle (°)	35.2 ± 6.8	0.446	0.000 **	-		−0.233	0.027 *
Lower angle (°)	38.0 ± 9.1	0.752	0.000 **	−0.233	0.027 *	-	

* *p* < 0.05; ** *p* < 0.01.

**Table 4:** Associations between basic attributes, thoracic spine angles, trunk angles, and medical checks.

	Physical Findings				Ultrasound Findings			
	Positive	Negative			Positive	Negative		
	*M* ± *SD*	*M* ± *SD*	*t*	*p*	*M* ± *SD*	*M* ± *SD*	*t*	*p*
Basic attributes								
Age (months)	137.5 ± 5.6	138.0 ± 5.0	−0.33	0.744	139.0 ± 5.0	136.6 ± 4.9	2.27	0.026 *
Number of years of competitive baseball	3.9 ± 1.0	3.8 ± 1.1	0.01	0.995	3.8 ± 1.0	3.9 ± 1.2	−0.13	0.990
Number of years pitching	1.9 ± 0.95	2.0 ± 1.1	−0.24	0.811	1.9 ± 1.1	2.1 ± 1.0	−0.68	0.496
Height (cm)	143.9 ± 6.6	144.8 ± 7.4	−0.38	0.704	145.8 ± 7.2	143.1 ± 7.1	1.75	0.084
Weight (kg)	37.1 ± 8.4	36.9 ± 6.4	0.08	0.939	37.9 ± 6.5	35.6 ± 6.8	1.63	0.106
Static standing thoracic angles								
Kyphosis angle (°)	26.7 ± 9.5	30.1 ± 7.6	1.42	0.159	28.5 ± 8.8	31.1 ± 6.3	1.52	0.133
Thoracic spine angle Th1 (°)	16.5 ± 7.3	18.3 ± 5.6	−0.98	0.332	17.5 ± 6.2	18.8 ± 5.4	−1.04	0.301
Thoracic spine angle Th12 (°)	10.2 ± 6.2	11.8 ± 5.0	−1.06	0.294	11.0 ± 5.2	12.3 ± 5.0	−1.13	0.263
Standing trunk retroflexion angles								
Overall angle (°)	74.2 ± 9.0	73.1 ± 10.2	0.39	0.700	71.4 ± 10.3	75.7 ± 9.2	−2.05	0.043 *
Upper angle (°)	33.0 ± 6.9	35.6 ± 6.8	−1.27	0.209	34.2 ± 7.2	36.4 ± 6.1	−1.54	0.128
Lower angle (°)	41.2 ± 8.2	37.5 ± 9.2	1.37	0.173	37.1 ± 8.7	39.2 ± 9.6	−1.08	0.284

* *p* < 0.05.

## Data Availability

The datasets used and/or analyzed during the current study are available from the corresponding author on reasonable request.
